# Allocation of Interferon Gamma mRNA Positive Cells in Caecum Hallmarks a Protective Trait Against Histomonosis

**DOI:** 10.3389/fimmu.2018.01164

**Published:** 2018-05-28

**Authors:** Fana Alem Kidane, Taniya Mitra, Patricia Wernsdorf, Michael Hess, Dieter Liebhart

**Affiliations:** ^1^Clinic for Poultry and Fish Medicine, Department for Farm Animals and Veterinary Public Health, University of Veterinary Medicine Vienna, Vienna, Austria; ^2^Christian Doppler Laboratory for Innovative Poultry Vaccines, University of Veterinary Medicine Vienna, Vienna, Austria

**Keywords:** *Histomonas meleagridis*, histomonosis, vaccination, interferon gamma, interleukin-13, *in situ* hybridization, immunofluorescence, image analysis

## Abstract

Histomonosis is a parasitic disease of gallinaceous birds characterized by necrotic lesions in cacum and liver that usually turns fatal in turkeys while it is less severe in chickens. Vaccination using *in vitro* attenuated *Histomonas meleagridis* has been experimentally shown to confer protection against histomonosis. The protective mechanisms that underpin the vaccine-induced immune response are not resolved so far. Therefore, the actual study aimed to evaluate the location and quantitative distribution patterns of signature cytokines of type 1 [interferon gamma (IFN-γ)] or type 2 [interleukin (IL)-13] immune responses in vaccinated or infected hosts. An intergroup and interspecies difference in the spatial and temporal distribution patterns of cytokine mRNA positive cells was evident. Quantification of cells showed a significantly decreased percentage of IFN-γ mRNA positive cells at 4 days post-inoculation (DPI) in caeca of turkeys inoculated exclusively with the attenuated or the virulent inocula, compared to control birds. The decrement was followed by a surge of cells expressing mRNA for IFN-γ or IL-13, reaching a peak of increment at 10 DPI. By contrast, turkeys challenged following vaccination showed a slight increment of cecal IFN-γ mRNA positive cells at 4 DPI after which positive cell counts became comparable to control birds. The increase in infected birds was accompanied by an extensive distribution of positively stained cells up to the muscularis layer of cecal tissue whereas the vaccine group maintained an intact mucosal structure. In chickens, the level of changes of positive cells was generally lower compared to turkeys. However, control chickens were found with a higher percentage of IFN-γ mRNA positive cells in cecum compared to their turkey counterparts indicating a higher resistance to histomonosis, similar to the observation in immunized turkeys. In chickens, it could be shown that the changes of cytokine-positive cells were related to variations of mononuclear cells quantified by immunofluorescence. Furthermore, gene expression measured by reverse transcription quantitative real time PCR confirmed variations in organs between the different groups of both bird species. Overall, it can be concluded that a proportionally increased, yet controlled, allocation of IFN-γ mRNA positive cells in caeca hallmarks a protective trait against histomonosis.

## Introduction

Histomonosis (syn.: blackhead disease, enzootic typhlohepatitis) is a parasitic disease of gallinaceous birds caused by the flagellate *Histomonas meleagridis* (1). The disease can cause high mortality rates in turkeys (*Meleagris gallopavo*) while in chickens (*Gallus gallus*) clinical signs are less severe, however, the infection entails an impaired performance in chickens leading to severe economic losses (2, 3). In both species, the parasite is able to infiltrate the cecum and triggers severe inflammation, thickening of the cecal wall and formation of fibrinous exudates. Histomonads can then migrate to the liver leading to multifocal areas of inflammation and necrosis, which is commonly observed in infected turkeys (4). Recently, a live vaccine prepared from an *in vitro* passaged clonal culture of *H. meleagridis* has been experimentally shown as safe and capable of providing protection from the disease (5–7). However, investigations on the immune response against histomonosis are rare and relevant protective mechanisms are so far not identified.

Only a few studies investigated the immune system of poultry in response to virulent *H. meleagridis* (8). It could be demonstrated that the level of serum antibody in turkeys may not be a key component in the protection against the parasite as passive immunization using immune sera or active immunization with killed vaccines failed to protect turkeys during challenge (5, 9, 10). However, local antibodies were measured in different parts of the intestine of infected chickens, a host species more resistant to fatal histomonosis, leading to the speculation that mucosal antibodies might be components that contribute toward protection (11). In a different study, an early onset of cytokine expression in cecal tonsils of infected chickens compared to infected turkeys was demonstrated and it was hypothesized to induce a timely immune response in order to prevent the parasite from migrating to the liver (12). Recently, flow cytometry was applied and changes in various immune cell populations in cecum, liver, spleen and blood of vaccinated and/or infected turkeys and chickens were determined showing a less pronounced changes in the frequency of immune cells in vaccinated hosts compared to an exacerbated influx in infected hosts (13).

Successful immunization depends on the ability to induce response selectively and reliably, and for this type 1/type 2 classes of immunity have been characterized in mammals in association with protection following infection or vaccination (14). Type 1/type 2 immunity has also been shown in chickens (15, 16) and can serves as a model for characterizing and optimizing immune responses in poultry for a better conferment of resistance through rational vaccine design (17). Previous studies on type 1/type 2 immunity following histomonosis, however, yielded inconclusive results. Powell et al. (12) found indications for a type 2 response with a persistently enhanced expression of interleukin (IL)-13 mRNA in infected chickens and turkeys, whereas a type 1 driven response with an augmented expression of interferon gamma (IFN-γ) mRNA was described when chickens were co-infected with histomonads and the intestinal worm *Heterakis gallinarum* (18). However, these studies utilized different infection protocols and applied undefined inocula for infection and the relevant pathway elicited during vaccination with attenuated live histomonads has never been investigated.

Moreover, in characterizing the expression patterns of cytokines of type 1/type 2 immunity, the widely utilized method has been reverse transcription quantitative real time PCR (RT-qPCR). While the technique delivered valuable information on the overall changes of transcription levels in homogenized samples, parameters such as the location and quantity of the expressing cells in tissues remained unlocked. Thus, to localize avian cells expressing T helper (Th)1/Th2 signature cytokines, respectively, INF-γ and IL-13, this study employed an *in situ* hybridization (ISH) approach to localize the cells in cecum, liver and spleen of vaccinated and/or infected turkeys and chickens. For verification of variations of the cytokines between the groups and species, RT-qPCR was employed to measure transcripts of IFN-γ and IL-13 mRNA on selected samples of turkeys and chickens, considering the different approach of both techniques. Furthermore, identification of immune cells recruited during vaccination or infection was conducted using immunofluorescence (IF) to reveal changes in populations of B cells, T cells and macrophage/monocytes in selected samples from chickens.

## Materials and Methods

### Animal Trial

#### Inoculum for Vaccination and Infection

The inocula used for vaccination or infection originated from a clonal culture of *H. meleagridis*/Turkey/Austria/2922-C6/04, established from cecal contents of a diseased turkey (19). The clonal culture was propagated for a short period of time (21 passages) to preserve virulence, hence used for challenge, or for 295 passages to achieve attenuation, according to previous protocols (5, 20). The parasites were kept as cryopreserved cultures (stored at −150°C) were then thawing and incubated at 40°C in a culture medium consisting of Medium 199 supplemented with Earle’s salts, l-glutamine, 25 mM HEPES and l-amino acids (Gibco™, Invitrogen, Lofer, Austria), 15% of fetal calf serum (Gibco™) and 0.66 mg of rice starch (Sigma–Aldrich, Vienna, Austria). Before administration, the number of viable cells was determined using a Neubauer cell counting chamber (Reichert, Buffalo, NY, USA) after staining with 0.4% trypan blue solution and adjusted to 1 × 10^6^ histomonads per ml of medium.

#### Birds

A total of 60 turkeys (B.U.T. 6™; Aviagen Turkeys Ltd., Tattenhall, UK) and the same number of specific pathogen-free chickens (VALO Biomedia, Osterholz-Scharmbeck, Germany) were used. The birds were numerically labeled with a subcutaneously attached tag and housed on deep litter. Unmedicated commercial turkey, respectively, chicken starter feed and water were provided *ad libitum*, except for a 5 h abstinence from feed directly after inoculation. The animal trials were approved by the institutional ethics committee and the national authority according to §26 of the Law for Animal Experiments, Tierversuchsgesetz—TVG (license number bmwf GZ 68.205/0147-II/3b/2013).

#### Experimental Design and Inoculation

In the main experiment (trial 1, Table 1), four groups of turkeys, as well as the same number of chicken groups, were arranged in separate groups (*n* = 15 per group). The groups comprised of only vaccinated turkeys or chickens (VT and VC), only IT and IC, vaccinated and infected turkeys or chickens (VIT and VIC) and control turkeys or chickens (CT and CC). Birds in group VIT and VIC were vaccinated at their first day of life. The challenge of the VIT and VIC was given on their 28th day of life, together with the infection or vaccination of the other groups.

**Table 1:** Experimental scheme and sampling strategy for different groups of turkeys and chickens inoculated with attenuated and/or virulent histomonads.

	Age of birds in days/days post-inoculation						
Group	1	28	32/4	35/7	38/10	42/14	49/21
Vaccinated turkeys (VT)		Vaccination	x^a^	x	X	X	x
Vaccinated chickens (VC)		Vaccination	x	x	X	X	x
Infected turkeys (IT)		Infection	x	x	X	N/A^b^	N/A
Infected chickens (IC)		Infection	x	x	X	X	x
Vaccinated and infected turkeys (VIT)	Vaccination	Infection	x	x	X	X	x
Vaccinated and infected chickens (VIC)	Vaccination	Infection	x	x	X	X	x
Control turkeys (CT)		Mock infection	x	x	X	X	x
Control chickens (CC)		Mock infection	x	x	X	X	x

*^a^Necropsy and sampling of three birds*.

*^b^Not applicable due to death caused by virulent histomonads at earlier time points*.

Each bird received a dosage of 6 × 10^5^ attenuated or virulent histomonads in 0.6 ml of medium, equally split *via* oral and cloacal application. Control birds were sham inoculated with equal volumes of pure culture media only. The oral and cloacal administration was performed using crop tubes attached to a sterile syringe, respectively, pipette tips on a 1 ml pipette. The feed was withdrawn for 5 h following inoculation to promote an adequate colonization of the parasite in the gut. To confirm successful inoculation, cloacal swabs were taken from every bird in intervals of 2–3 days and parasites were incubated for 3 days at 40°C in a culture medium mentioned above. Growth was controlled by microscopical examination of viable parasites.

In a similar setting, an additional experiment (trial 2) was conducted for generating additional cecum, liver and spleen samples for IF labeling of B cells, T cells and monocytes/macrophages in the above-described organs of chickens. For that, three groups of chickens were used: IC, VC, and control chickens (CC). Each infected, respectively, vaccinated bird received 1 × 10^4^ histomonads orally and cloacally.

#### Clinical Monitoring and Postmortem Lesion Scoring

The birds were monitored daily for clinical signs of histomonosis such as ruffled feathers, depression and yellowish diarrhea. Birds showing clinical signs of the disease were euthanized.

At 4, 7, 10, 14 and 21 days post-inoculation (DPI), three predefined birds per group were killed according to their tag-numbers by bleeding after intravenous injection of sodium thiopental (Sandoz, Kundl, Austria). Postmortem examination and grading of lesions in cecum and liver were performed according to a previous scheme that used scores from 0 (no lesion) to 4 (severe lesions) (11, 21).

### Histological Techniques

#### ISH for Localization of *H. meleagridis*

Pieces of mid-cecum, liver and spleen samples intended for ISH were fixed in 4% neutral buffered formaldehyde (SAV liquid production, Flintsbach, Germany) for at least 48 h at room temperature. The tissues were then trimmed and rinsed in running tap water for an hour before proceeding to routine histological processing. The processing involved dehydration in serially graded alcohols (80, 96 and 100%), clearing in Neo-Clear^®^ (Merck, Darmstadt, Germany) and paraffin infiltration. Finally, the tissues were embedded in paraffin wax and stored until use. For the scheduled ISH, serial sections of 4 µm were prepared using Microme HM360 rotary microtome (Microme, Walldorf, Germany) and mounted on Superfrost Plus slides (Menzel-Gläser, Braunschweig, Germany). To further ensure adhesion of sections on the slides, an overnight treatment of the slides was allowed at 37°C. The ISH with *H. meleagridis* specific digoxigenin (DIG)-labeled oligonucleotide probes was performed according to a published protocol (22).

#### ISH for Localization of IFN-γ or IL-13 mRNA

##### Generation of RNA Probes

The use of *in vitro* transcribed DIG-labeled RNA probes for detection of chicken IFN-γ and IL-13 mRNA in liver and spleen paraffin sections has been established earlier. The same approach was followed to establish turkey’s staining antisense IFN-γ or IL-13 RNA probes together with negative control sense probes as previously described (23). Briefly, gene-specific primers were designed to amplify the mRNA of turkey IFN-γ and IL-13 (Table 2). The targets were amplified from total RNA extracted from the spleen of a non-infected turkey by RT-PCR using QIAGEN OneStep RT-PCR kit (Qiagen, Hilden, Germany). The PCR products were then separated on 1.5% Agarose gel and purified using QIAquick^®^ Gel Extracti*o*n Kit (Qiagen). The respective cDNAs were ligated into pCR^®^4-TOPO^®^ vector for cloning in One Shot^®^ TOP10 Chemically Competent *E. coli*, according to the manufacturer’s instructions (Invitrogen, Carlsbad, CA, USA). The nucleotide sequence and insertion of the cloned products were verified by sequencing (LGC Genomics, Augsburg, Germany).

**Table 2:** Primers, Genbank accession number and the size of the PCR product used for RNA probe generation for *in situ* hybridization of interferon gamma (IFN-γ) and interleukin (IL)-13 mRNA.

Target	Primer sequence	Accession number	Product size (bp)
Turkey-IFN-γ	F: CACCAAGAAGATGACTTACCAG R: TGAGGATGGCTCCTTTTCC	XM_003202048	486
Turkey-IL-13	F: GCTCCATGCCCAAGATGAAG R: AGCGTTGGCAAGAAGTTCC	AM493431	289

Plasmids validated to contain the cDNA of the cytokines were linearized with *Spe*I or *Not*I restriction enzymes (Thermo Scientific, Dreieich, Germany). Depending on the insertion, T7 or T3 RNA polymerases were used separately to generate sense or antisense DIG-labeled RNA probes using DIG RNA Labeling Kit (SP6/T7) (Roche Diagnostics, Mannheim, Germany) according to the manufacturer’s protocol. The probe yield was quantified using NanoDrop spectrophotometer (Thermo Scientific) and the size of the transcribed probes was verified by gel electrophoresis. Furthermore, the efficiency of the DIG-labeling was assessed by immunoblotting dilution series of DIG-labeled RNA probes according to the manufacturer’s recommended protocol given in the DIG RNA Labeling Kit (SP6/T7) (Roche Diagnostics) with some modifications mentioned in the previous work (23).

##### RNA ISH

The ISH protocol for staining cytokine mRNA positive cells was described in an earlier study (23). Briefly, paraffin sections were deparaffinized with Neo-Clear^®^ (Merck) and rehydrated in a series of graded alcohols (100, 96 and 70%) and diethyl pyrocarbonate (DEPC) treated distilled water. The sections were then digested with 2.8 µg/ml of proteinase K (Invitrogen, Carlsbad, CA, USA) in 0.05 M Tris–HCl (pH 7.5), at 40°C for 30 min. Afterwards, rinsing and dehydration were performed in DEPC-treated water and increasing concentrations of ethanol. Following a short period of air drying, the sections were covered with a hybridization mix composed of 50% formamide, 4× standard saline citrate buffer (SSC), Herring’s Sperm DNA (500 ng/ml), 1× Denhardt’s solution, 10% dextran sulfate, and the optimal concentration of the respective DIG-labeled RNA probe. A concentration of probes that ranged from 10 to 1,000 ng/ml of hybridization buffer was tested initially to find a defined concentration for further use. Hybridization of the probe with the target RNA was performed in a humidified chamber for overnight at 40°C.

On the next day, stringency washes were made with 2× SSC at room temperature for 30 min, followed by RNase A (20 ng/ml) (Roche Diagnostics) treatment in a solution mix of 0.5 M NaCl, 5 mM Tris–HCl (pH 7.5) and 1 mM EDTA, at 40°C for 30 min. Further washing steps were done two times in 1× and 0.1× SSC at room temperature for 10 min each.

For conjugating the DIG-labeled hybrid-complexes with an enzyme to be used for the color reaction, the sections were first incubated in a blocking solution (50% of Buffer I, 0.3% TRITON^®^-X and 5% normal goat serum in distilled water) for 30 min in a humidified chamber. The blocking solution was replaced by anti-DIG-AP-antibody diluted 1:100 in the blocking solution mix and incubated for 1 h at room temperature. Afterward, washing and equilibration were performed in three steps. First, unbound antibody was removed by washing the slides in a 1 to 1 dilution of Buffer I (100 mM Tris–HCl pH 7.5, 150 mM NaCl) in distilled water for 15 min. Second, the washing mentioned before was repeated and finally, equilibration in Buffer II (100 mM Tris–HCl pH 7.5, 100 mM NaCl, 50 mM MgCl_2_, final pH 9.5) was made for 10 min.

For visualizing the target molecules, color reaction was developed using a substrate mix of an equilibration buffer (Buffer II), NBT (4-nitro blue tetrazolium chloride) (0.45 mg/ml), BCIP (5-bromo-4-chloro-3-indolyl-phosphate) (0.175 mg/ml) (Roche Diagnostics) and Levamisole (240 µg/ml) (Sigma, Deisenhofen, Germany). The color reaction was performed in a humidified chamber under dark conditions for overnight. On the next day, the reaction was terminated by immersing the sections in Tris-EDTA (pH 8.0) for at least 10 min. Finally, the sections were counterstained with Gill’s hematoxylin (Merck) and mounted under coverslips (Menzel-Gläser) with Aquatex^®^ aqueous mounting medium (Merck). For controlling the specificity of the technique, ISH was performed using the respective sense probes. Moreover, a slide from sections incubated with DEPC-water instead of the probes was used in parallel as negative control.

#### Immunofluorescence

Cecum, liver and spleen of one bird per group obtained from trial 2 were cryopreserved after embedding in Tissue-Tek^®^ (Sakura Finetek Europe B.V., Flemingweg, the Netherlands) on aluminum foils. Liquid nitrogen was used to freeze the embedded tissue samples. The frozen samples were then stored at −80°C until they were cut into 5 µm slices by a cryostat (CM1800, Leica Mikrosysteme GmbH, Vienna, Austria) and directly processed for IF.

A multicolor staining protocol was employed using mouse anti-chicken Bu-1-Biotin for the detection of B cells, unlabeled mouse anti-chicken CD3 for the detection of T cells and mouse anti-chicken KUL01-PE for the detection of monocytes/macrophages (all SouthernBiotech, Birmingham, AL, USA). For labeling, the cryosections were first fixed in pre-cooled acetone at −20°C. After drying and rehydration in PBS, they were treated with blocking solution (PBS, 2% bovine serum albumin and 10% goat serum) and then incubated overnight with the CD3 antibody at 4°C in a humidified chamber.

On the next day, slides were repeatedly washed with PBS and covered with the secondary antibody goat anti-mouse Alexa 488 (Invitrogen, Vienna, Austria). Following extensive washing and treatment with the blocking solution mentioned above, the other two primary antibodies (biotin-labeled anti-chicken Bu-1 and anti-chicken KUL01 directly labeled with r-phycoerythrin) were applied on the tissues and incubated overnight in a humidified chamber in the same way as mentioned before.

On the third day, streptavidin-Alexa 647 (Life Technologies, Vienna, Austria) was used for conjugation with the Bu-1 antibody. Cell nucleus staining was performed with 4, 6-diamidine-2-phenylindole dihydrochloride (DAPI) (Boehringer Mannheim GmbH, Germany). An isotype control for IgG1k (eBioscience, Vienna, Austria) together with the goat anti-mouse Alexa 488 antibody and samples without primary antibodies were included as controls to confirm the specificity of primary antibody binding.

#### Microscopy and Analysis of Images for Quantification of Stained Cells

Tissue sections processed by chromogenic ISH for staining cytokine mRNA expressing cells were examined using light transmission microscopy under Zeiss Axio Imager Z1 (Carl Zeiss Microscopy GmbH, Germany). Sections were examined for the location of the stained cells in context of the histological architecture of the respective tissues. Afterward, digital images of the sections were made using a USB camera and the TissueFAXs image acquisition software (TissueGnostics GmbH, Vienna, Austria). The digital images were analyzed for quantification of cells by HistoQuest (version 4.04.0150, TissueGnostics GmbH). For cell quantification, a region of interest (ROI) was selected randomly excluding artifacts, major blood vessels and edges of the sections. The ROI for each sample was a total tissue area of 2 mm^2^ divided among equal sizes of eight rectangular ROI. Then, a total number of cells within those 2 mm^2^ of the ROI were counted based on their blue nuclei staining after using hematoxylin as a master channel. Valid cells were gated by hematoxylin area versus hematoxylin mean intensity. By applying the software integrated color separation method named single reference shade, a second color detection was performed to automatically separate the hematoxylin (blue) nuclei staining from NBT-BCIP (dark violet) staining based on their optical density. Cutoff values for discriminating NBT-BCIP positive cells from negative cells were set interactively based on the background signal measured in the negative controls (sections treated with sense probes or no probe). In addition, backward viewing was performed to verify the correct discrimination of NBT-BCIP stained cells from negative cells by the source image, shades and color overlay. The percentage of NBT-BCIP stained cells out of the total quantified cells in each tissue section was then exported to SPSS (SPSS 21.0, IBM, Armonk, NY, USA) for statistical analysis.

The procedure for quantification of cells in triple stained IF sections were described earlier (24). Briefly, a multichannel acquisition was made using a Zeiss Axio Imager Z1 fitted with a PCO USB camera and eight regions of interest were selected in digital images of each tissue sample covering an area of 2 mm^2^. The identification of each cell was achieved by TissueQuest software using the fluorescence of DAPI (master channel). Valid cells were gated by DAPI area versus DAPI mean intensity. From this population of cells, T cells, B cells and monocytes/macrophages with their respective fluorescence signals were subsequently gated. According to the setup of the respective gates, T cells, B cells and monocytes/macrophages were quantified by the software.

### RT-qPCR

Stabilization of RNA in pieces of cecum, liver and spleen samples was performed by immersing the tissues in RNAlater^®^ solution (Qiagen) according to the manufacturer’s instruction. Total RNA was then extracted using RNeasy^®^ mini kit (Qiagen) after homogenizing the tissues with QIAshredders (Qiagen) according to manufacturer’s instructions. Every RNA sample was assessed for purity, quantity and integrity by using NanoDrop 2000 (ThermoFisher Scientific, Vienna, Austria) and 4200 TapeStation (Agilent Technologies, Waldbronn, Germany), respectively. The RNA samples were stored at −80°C until used.

The expression level of IFN-γ or IL-13 mRNA was quantified by applying TaqMan one-step real-time RT-qPCR using Brilliant III Ultra-Fast QRT-PCR master mix kit (Agilent Technologies, Waldbronn, Germany). Previously published primers and probes for reference genes TFRC (transferring receptor protein 1) and RPL13 (ribosomal protein L13) for turkeys or RPL13 and TBP (TATA box binding protein) for chickens, as well as for the mentioned cytokines were applied (Table 3) (12, 25). Amplification of the primary transcripts and quantification of their specific products were performed using AriaMx real-time PCR system (Agilent Technologies, Waldbronn, Germany) together with the Agilent AriaMx1.0 software (Agilent Technologies, Waldbronn, Germany). The thermal cycle profile was as follows: 1 cycle of RT at 50°C for 10 min followed by 95°C for 3 min to hot start, 40 cycles of amplification at 95°C for 5 s and 60°C for 10 s. Different types of controls such as non-reverse transcriptase and non-template control were run to check for genomic DNA contamination and overall PCR contamination.

**Table 3:** Reverse transcription-qPCR primers and probes.

Target	Primer and probe sequences	Reference
Turkey IFN-γ	F: AACCTTCCTGATGGCGTGAA R: CTTGCGCTGGATTCTCAAGTC P: HEX-AAAGATATCATGGACCTGGCCAAGCTTCA-BHQ1	(12)
Turkey IL-13	F: CCTGCACGGCCAGATGA R: GGCAAGAAGTTCCGCAGGTA P: CY5-TGCCAGCTGAGCACCGACAACG-BHQ1	
Chicken IFN-γ	F: GTGAAGAAGGTGAAAGATATCATGGA R: GCTTTGCGCTGGATTCTCA P: HEX-TGGCCAAGCTCCCGATGAACGA-BHQ1	
Chicken IL-13	F: CACCCAGGGCATCCAGAA R: TCCGATCCTTGAAAGCCACTT P: CY5-CATTGCAAGGGACCTGCACTCCTCTG-BHQ1	

RPL13	F: GGAGGAGAAGAACTTCAAGGC R: CCAAAGAGACGAGCGTTTG P: HEX-CTTTGCCAGCCTGCGCATG-BHQ1	(24)
TBP	F: CTGGGATAGTGCCACAGCTA R: GCACGAAGTGCAATGGTTT P: ROX-TGCAACCAAGATTCACCGTGGA-BHQ2	
TFRC	F: AGCTGTGGGTGCTACTGAA R: GGCAGAAATCTTGACATGG P: ROX-CTCTGCCATGCTGCATGCCA-BHQ2	

The cytokines’ average cycle threshold (Ct) values for every organ were normalized using geometric mean Ct of the reference genes, to exclude technical variations during sampling and processes of RT-qPCR. Fold change of cytokines was then calculated from the normalized mean Ct value of cytokines from each group by applying the formula 2^(−ΔΔCt)^ (26).

### Statistical Analysis

The statistical package SPSS (SPSS 21.0, IBM, Armonk, NY, USA) was used to execute the descriptive and non-descriptive statistics. Mean lesion scores of cecum or liver were calculated for each group of turkeys or chickens necropsied at the specified DPI. Similarly, total mean lesion scores (MLSs) of cecum or liver were calculated for each group of turkeys or chickens by pooling the total lesion scores of each group and divided by the total number of birds in the group. For comparing differences in the mean percentage of cytokine mRNA positive cells, first normality of the data distribution obtained from percentages of quantified ISH-positive cells for each cytokine was tested using Kolmogorov–Smirnov test according to the tissue and host species. Then, since the data generated from quantification of turkey IFN-γ and IL-13 mRNA positive cells were normally distributed, mean values obtained from the different groups necropsied at different days were compared with the control using independent samples *t*-test. By contrast, due to lack of normality of data distribution in chicken tissues, Mann–Whitney *U* test was used to test the significance of mean differences. Furthermore, *t*-test was used to compare mean of normalized Ct values of the RT-qPCR data. Mean value differences were considered significant when the *P*-value was less than 0.05 (*P < 0.05).

## Results

### Animal Trial

Turkeys inoculated with virulent histomonads without prior vaccination (IT) exhibited typical signs of histomonosis such as a ruffled feather, depression and sulfur-colored diarrhea starting from 7 DPI. Consequently, two birds had to be euthanized due to severe suffering at 11 DPI while the remaining animals were found dead at 12 and 13 DPI. There were not any overt clinical signs in the rest of the turkeys’ as well as all of the chickens’ groups until the experiment was terminated at 21 DPI. Microscopical investigation of cultured cloacal swabs, however, confirmed the presence of the flagellate in the gut of birds of every group except the controls (data not shown).

**Figure 1:**
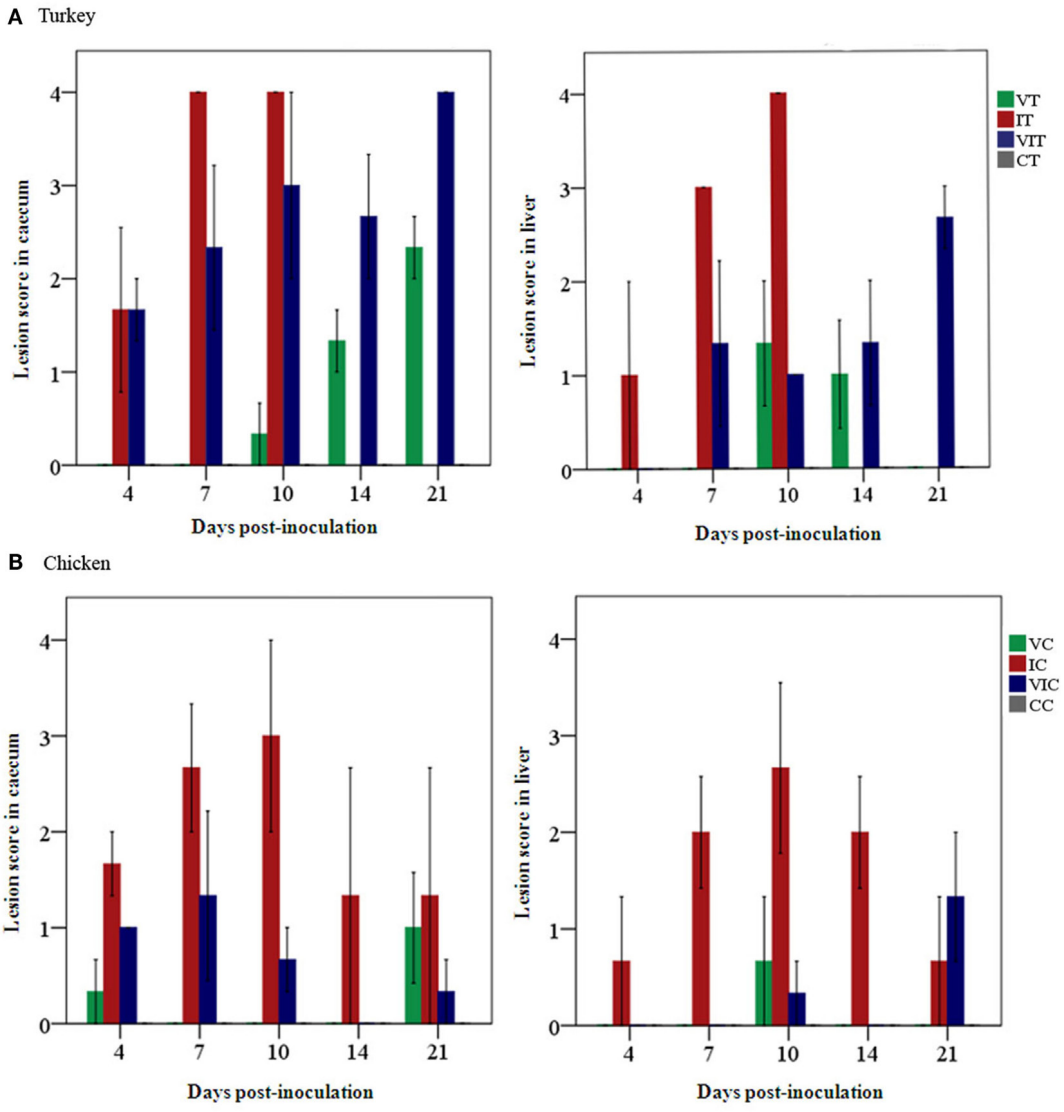
Mean lesion scores (*n* = 3 birds/day) recorded in cecum and liver. Scores were graded from 0 to 4, indicating no lesion to severe lesion in **(A)** groups of vaccinated turkeys (VT), infected turkeys (IT) and vaccinated and infected turkeys (VIT) and in **(B)** groups of vaccinated chickens (VC), infected chickens (IC), or vaccinated and infected chickens (VIC) necropsied at different days post-inoculation. There were no lesions observed in control turkeys or chickens (CT or CC) throughout the time points.

Necropsy findings are given by MLS of every group on each sampling day. Total mean lesions scores differed according to the given inoculum, respectively, were found to be reduced in birds that received the vaccination (Figure 1). In turkeys (Figure 1A), the global MLS was 0.8 in cecum and 0.5 in the liver of VT. Turkeys in group IT had a total MLS of 3.2 and 2.8 in cecum and liver, respectively. In VIT, a total MLS of 2.7 in cecum and MLS of 1.3 in liver were observed. In chickens (Figure 1B), the highest total MLS recorded was in cecum and liver of IC (2 and 1.6, respectively). Chickens in group VC and VIC displayed a global MLS below 0.5 in both cecum and liver. None of the control birds in group CT or CC showed lesions at any sampling day.

### Localization of *H. meleagridis* in Tissue Sections

Detailed results on frequencies of *H. meleagridis* localized by ISH in cecum and liver of the birds are given in Table 4. In cecum, histomonads could be localized in the majority of the turkeys that were inoculated either with the attenuated and/or virulent histomonads. Groups of chickens inoculated with histomonads showed the parasite localized in the cecal sections yet with less frequency than the turkeys inoculated with the same histomonads.

**Table 4:** Number of samples positive for *Histomonas meleagridis* detected by *in situ* hybridization (*n* = 3 birds/day).

		Vaccinated DPI					Infected DPI					Vaccinated and infected DPI				
		4	7	10	14	21	4	7	10	14	21	4	7	10	14	21
Turkey	Cecum	0	3	3	2	2	3	3	3	N/A	N/A	3	3	3	2	2
	Liver	0	0	0	0	1	3	3	3	N/A	N/A	0	1	1	0	1

Chicken	Cecum	0	1	2	0	0	1	2	2	1	1	2	1	1	2	0
	Liver	0	0	0	0	0	0	1	2	0	0	0	0	0	0	0

*DPI, days post-inoculation*.

The presence of histomonads was generally uncommon in livers except in IT in which the parasite was detected throughout the sampling days. The protozoa could be found in only one VT collected at 21 DPI and few liver samples (3 out of 15 birds) taken from VIT. In chickens, only three liver samples taken from IC were positive for histomonads.

**Figure 2:**
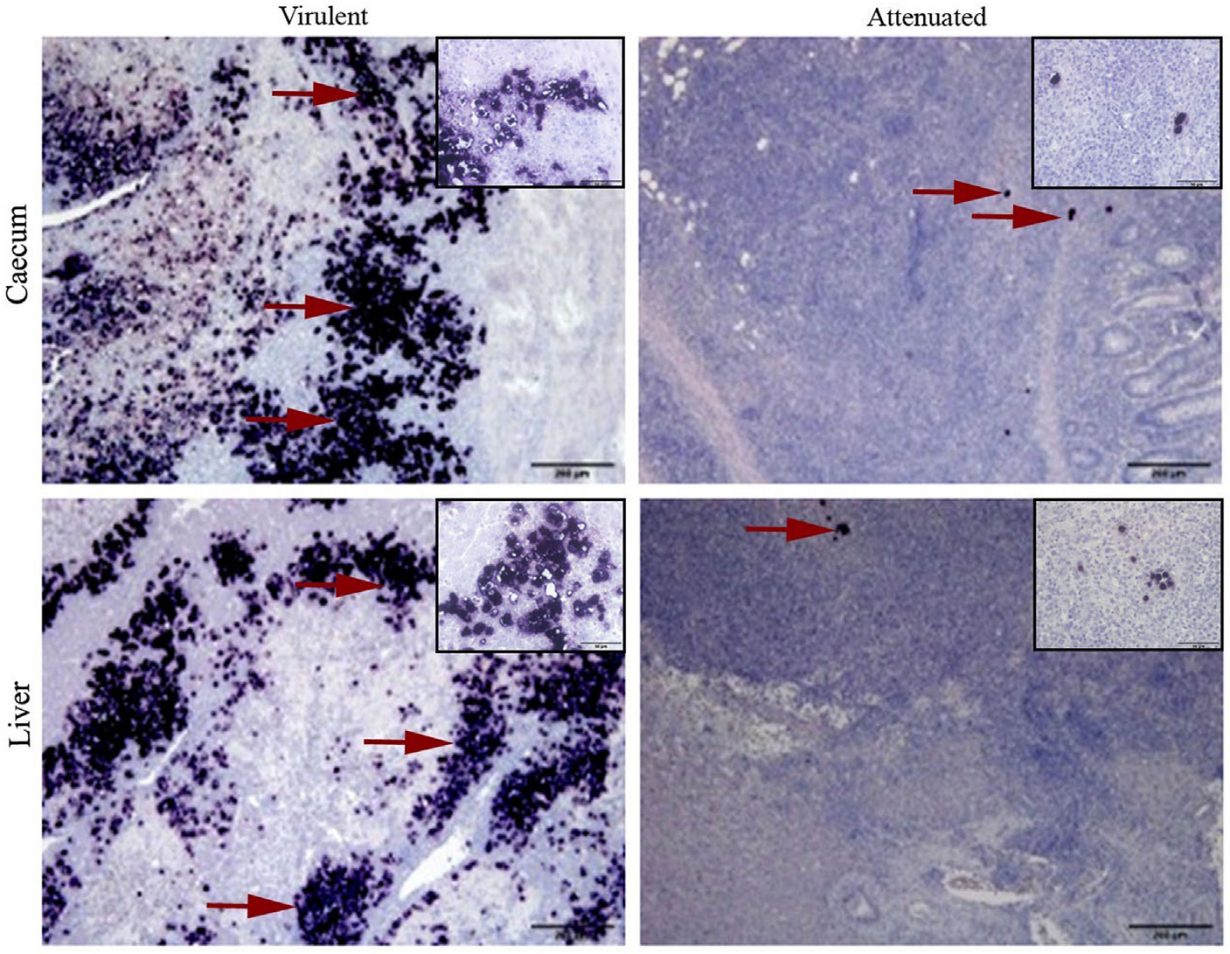
Virulent and attenuated histomonads localized in cecum and liver by *in situ* hybridization. Stained histomonads could be seen in dark purple (examples shown in red arrow). Virulent histomonads could be seen in larger number, whereas only a few attenuated histomonads could be detected surrounded by an influx of mononuclear cells. Samples are representatives for tissues collected from turkeys inoculated with virulent histomonads (group infected turkey) necropsied on 10 days post-inoculation (DPI) and turkeys inoculated with only attenuated histomonads (group vaccinated turkeys) necropsied on 21 DPI. Insets show higher magnifications of histomonads and the surrounding cells in the respective tissue sample.

The spleen of all turkey and chicken samples, independent of the group they belong to, were without parasite infestation and the control groups confirmed the status of non-inoculated birds. Localization and distribution of virulent or attenuated histomonads in cecum and liver are comparatively shown in turkeys of the respective groups (Figure 2).

### Evaluation of IFN-γ and IL-13 RNA Probes

The DIG-labeled RNA probes generated to hybridize to turkey IFN-γ or IL-13 mRNA in paraffin sections of cecum, liver and spleen could label cells containing the mRNA of the cytokines at a probe concentration of 600 or 300 ng/ml of hybridization buffer for IFN-γ or IL-13, respectively. Similarly, probes against chicken IFN-γ or IL-13 mRNA resulted in distinct coloration of target cells at a concentration of 400 or 100 ng/ml of hybridization buffer, respectively. Positive cells could be recognized by their dark purple color developing from the enzymatic reaction of NBT-BCIP. A higher or lower concentration of probes than the ones mentioned resulted in fainting or over staining of sections, hindering identification of individual cells (data not shown).

### Localization and Quantitative Distribution Patterns of IFN-γ or IL-13 mRNA Positive Cells Following Vaccination or Infection

#### Spatial and Temporal Distribution Differences of Quantified IFN-γ mRNA Positive Cells

**Figure 3:**
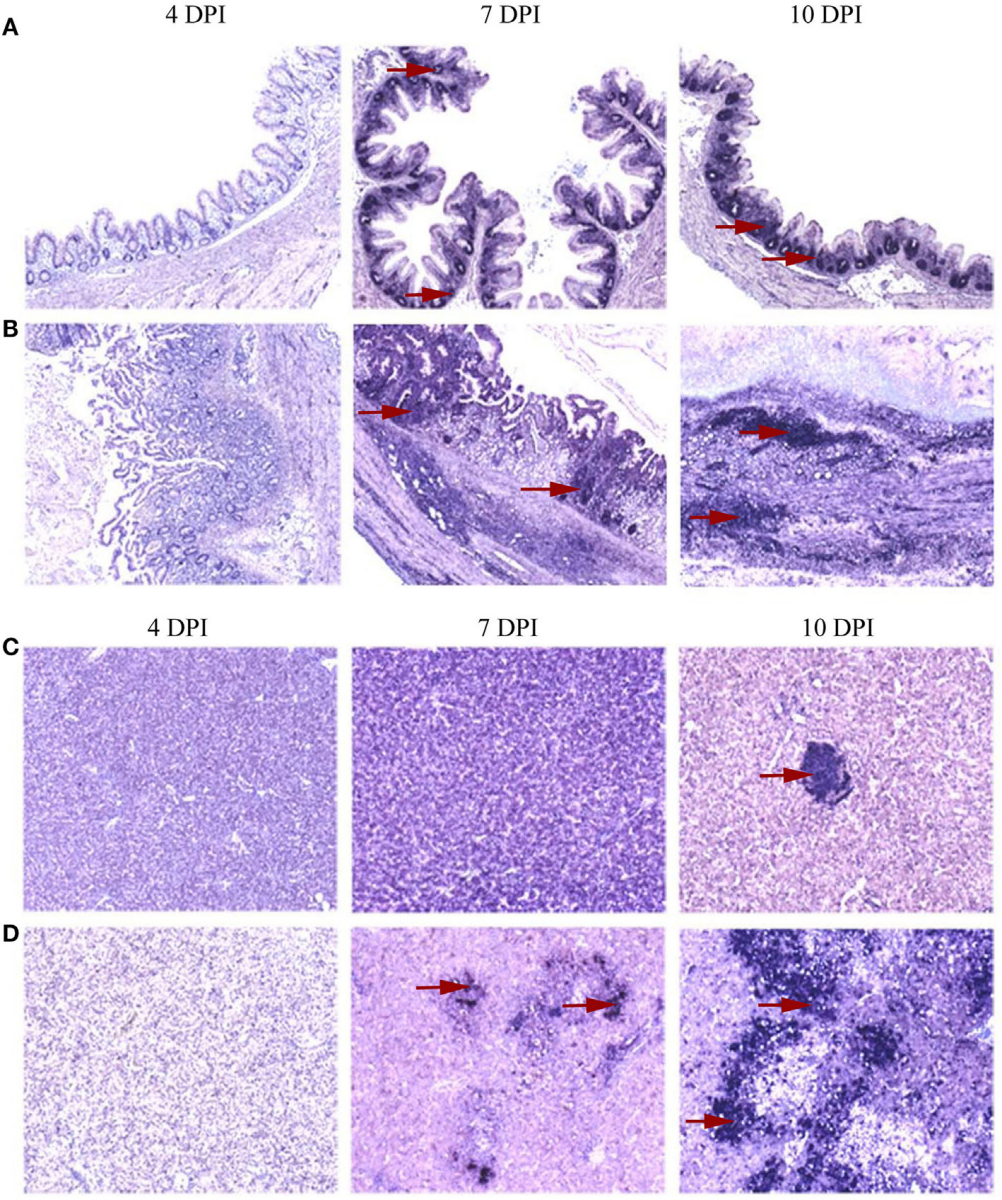
Distribution of interferon gamma (IFN-γ) mRNA positive cells localized by *in situ* hybridization in cecum and liver. A distinct distribution pattern of cytokine mRNA positive cells in the primarily affected organs was evident between groups of turkeys that received only attenuated parasites and those inoculated with virulent *Histomonas meleagridis*. Representative distribution patterns are given using IFN-γ mRNA positive cells with areas of positive cells indicated by red arrows. At 4 days post-inoculation (DPI), positive cells were rarely distributed in caeca which then became abundant at 7 and 10 DPI with accumulation of positive cells being restricted to the lamina propria of birds that receive the attenuated culture **(A)**, whereas virulent histomonads triggered further distribution of positive cells to the muscularis layer with losing of the mucosal fold at 10 DPI **(B)**. In livers, positive cells were occasionally found as sporadic cells studded between hepatocytes and lymphoid aggregates **(C)** or in more expansive distribution comprising mononuclear infiltrates when affected by virulent histomonads **(D)**.

**Figure 4:**
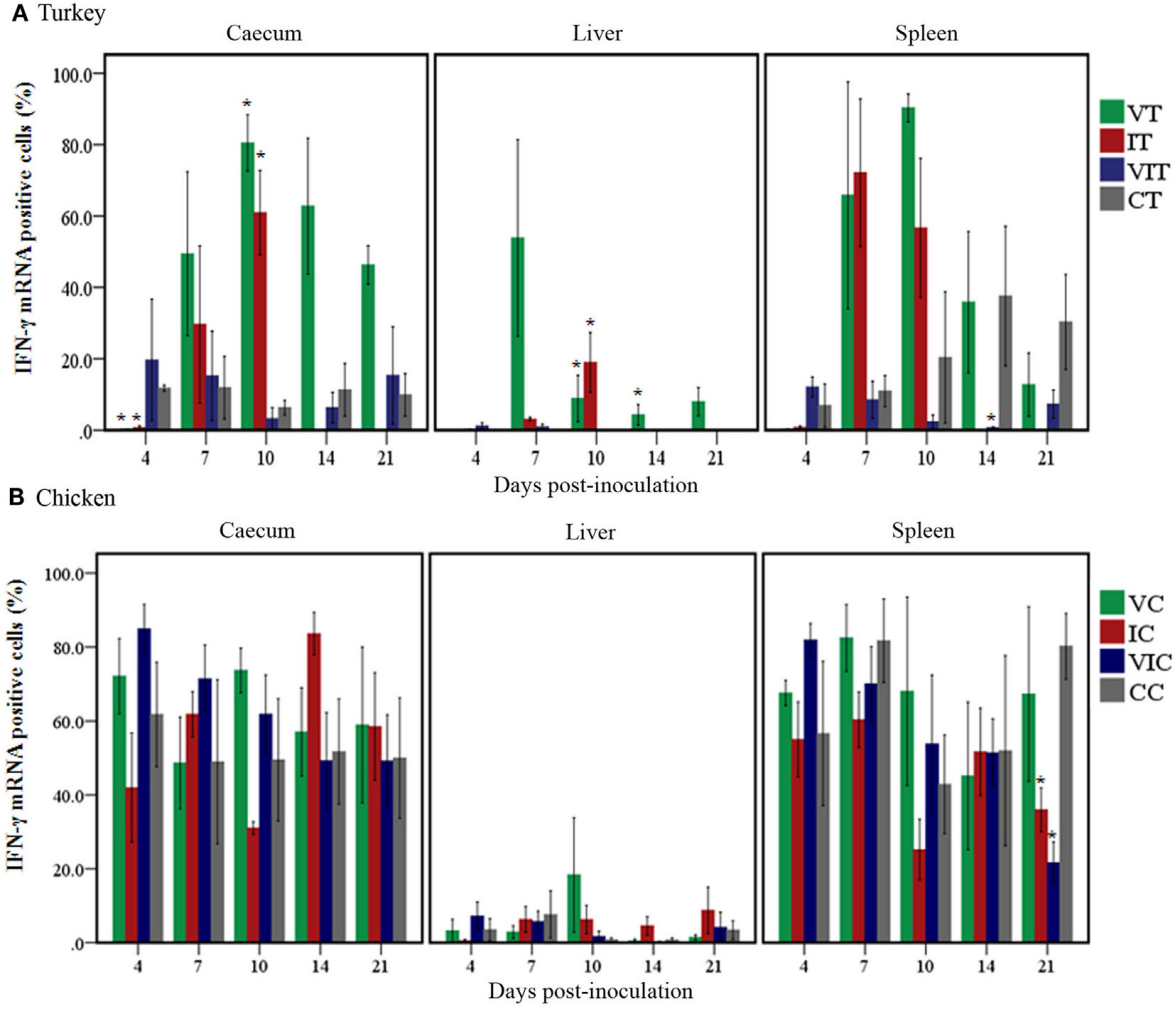
Quantity of interferon gamma (IFN-γ) mRNA positive cells. Positive cells were quantified by TissueFAXS and its accompanying software HistoQuest in cecum, liver and spleen of turkeys **(A)** and chickens **(B)** inoculated with attenuated and/or virulent *Histomonas meleagridis*. Mean values of percentages of mRNA positive cells in each treatment group were compared with the age-matched control at the respective days post-inoculation. *n* = 3 birds/day, *P < 0.05.

The spatial distribution patterns of stained cells in cecum and liver of turkeys inoculated with the tentative vaccine or the virulent inoculum are shown in Figure 3. In cecum, IFN-γ mRNA positive cells in VT were exclusively localized as individual cells studded to the intraepithelial region and as small aggregates of cells in the lamina propria as seen in control birds throughout the study period. Quantification of these cells (Figure 4A), however, revealed a significantly lower percentage of positive cells at 4 DPI. The early decrement in VT was followed by an abrupt increment characterized by a statistically significant change at 10 DPI. Relative numbers of IFN-γ mRNA positive cells gradually decreased on 14 DPI until the experiment ended at 21 DPI, although being still higher than in the control birds. In IT, the distribution pattern of positive cells at 4 DPI was similar to VT and significantly lower compared to the control group. At 7 and 10 DPI, positive cells showed an extensive spread within the lamina propria and muscularis, along with a total loss of the mucosal structure. Coincidentally, the relative number of these cells was significantly higher at 10 DPI. There were no surviving birds left in group IT from 14 DPI onward. In VIT, the distribution of positive cells started out like mentioned above for the other groups. However, at 4 DPI, the percentage of cells was slightly higher than in the control, albeit statistically not significant. From 7 DPI onward, the cecal wall had infiltrated mononuclear cells with IFN-γ mRNA positive cells sporadically distributed among them. Calculation of positive cells, however, was more or less similar to birds from the control group.

In the liver of VT and CT, stained cells could be found as lymphoid aggregates and sporadically between hepatocytes. There was no change in the spatial distribution until 21 DPI when two liver samples of VT showed a restricted yet ectopic lymphoid alteration in one area with few positive cells interspersed within. Despite the similarity in the spatial distributions of stained cells in these groups, relative numbers of cells (Figure 4A) were higher in VT from 7 DPI onward with significant changes observed at 10 and 14 DPI. In IT, changes, both in the location and percentage of positive cells, were different at and after 7 DPI when positive cells were observed within the inflammatory infiltrates between the parenchyma and periphery of necrotized regions. The percentage of these cells was also significantly higher at 10 DPI. Positive cells in livers of VIT were similar to CT, both in location and abundance.

In spleen, positive cells had a wider spatial distribution ranging from aggregates in the periarteriolar lymphatic sheath to solitary cells in the rest of white and red pulp, regardless of the experimental treatment. Percentages of positive cells (Figure 4A), however, significantly decreased in VIT at 14 DPI, whereas the changes in the other groups were not statistically supported.

The location of positive cells in cecum, liver and spleen of control and affected tissues described in turkeys also applies to chickens and differences among the groups of chickens were mostly marginal. In VC, however, there was neither a location nor a percentage change in the relative numbers of positive cells in caeca throughout the experiment (Figure 4B). In caeca of IC, positive cells extensively spread to the lamina propria as the disease progressed at and after 7 DPI. Similarly, in VIC, a localized yet infiltrated area with positive cells was observed only at 7 DPI. Livers of VC and VIC were similar to control birds throughout the experiment, whereas positive cells were observed in infiltrates of leukocytes of affected livers of IC collected at 14 and 21 DPI. In spleen, the only noticeable changes were the significantly decreased percentage of positive cells in the spleens of IC and VIC at 21 DPI.

#### Spatial and Temporal Distribution Differences of Quantified IL-13 mRNA Positive Cells

The localization of IL-13 mRNA positive cells in turkeys and chickens was similar to the distributional localization of IFN-γ mRNA positive cells described for each group and DPI. The percentage of positive cells, however, varied at different DPI.

**Figure 5:**
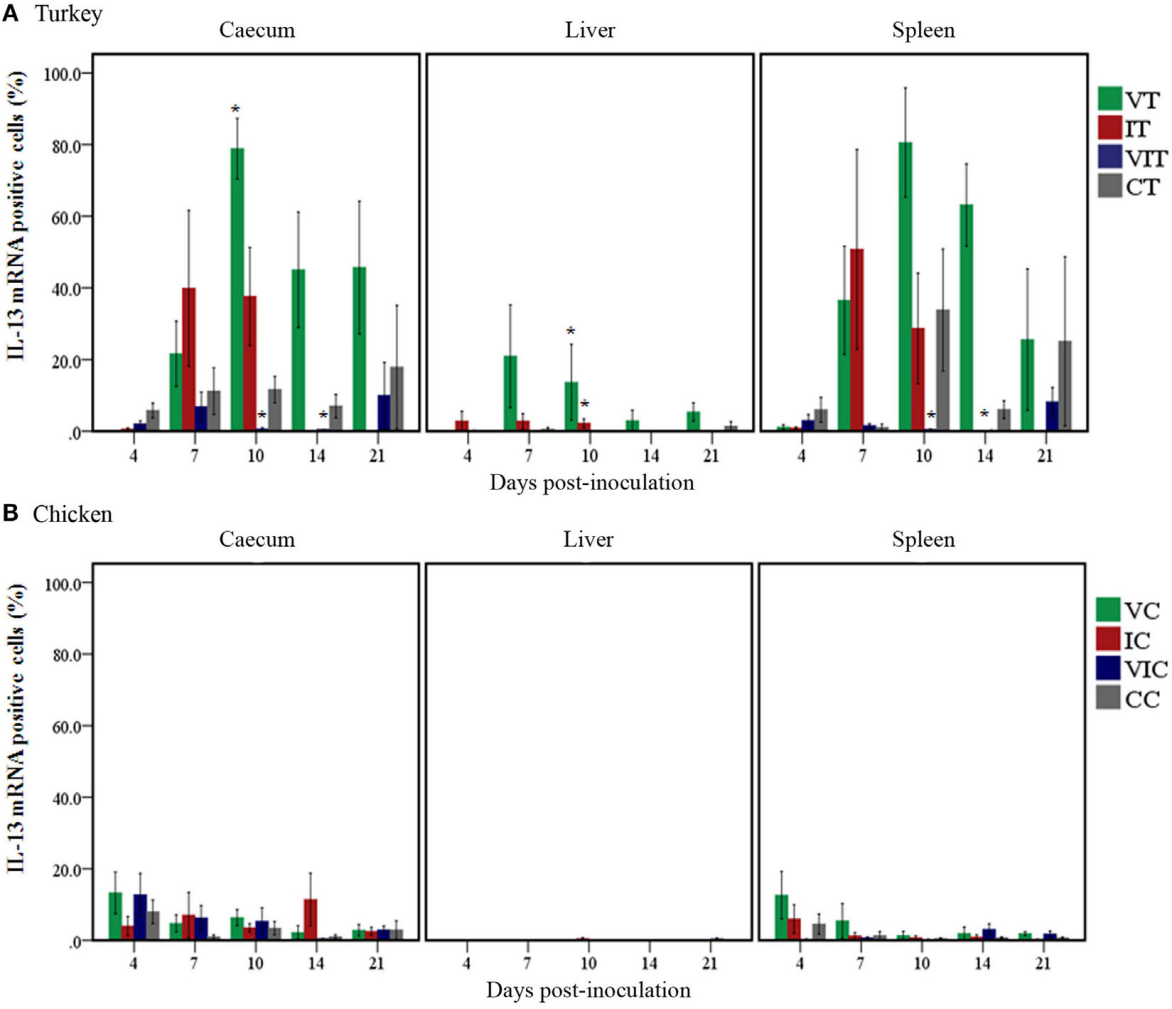
Quantity of interleukin (IL)-13 mRNA positive cells. Positive cells were quantified in caeca, livers and spleens of groups of turkeys **(A)** and chickens **(B)** inoculated with attenuated and/or virulent *Histomonas meleagridis*. Mean percentage obtained from each treatment group was compared with the age-matched control. *n* = 3 birds/day, *P < 0.05.

In turkeys (Figure 5A), the percentage of IL-13 mRNA positive cells increased in the cecum of VT from 7DPI onward; the only significant change, however, was the higher percentage at 10 DPI. Positive cells also increased in caeca of IT at 7 and 10 DPI; these changes nevertheless were not statistically significant. By contrast, the percentage of positive cells showed no change or was lower in VIT throughout the trial when positive cells significantly decreased at 10 and 14 DPI. In livers, the percentage of positive cells significantly increased in VT as well as IT at 10 DPI. In spleens, even though the percentages increased variably in VT and IT at and after 7 DPI, the changes were not statistically significant. By contrast, the percentage significantly decreased in spleens of VIT at 10 and 14 DPI.

In chickens, changes in the percentage of IL-13 mRNA positive cells (Figure 5B) were not significant, but slight changes could be noticed at 4 DPI when positive cells increased in the cecum of VC and VIC as well as in spleens of VC.

#### Comparison of Quantified IFN-γ or IL-13 mRNA Positive Cells

**Figure 6:**
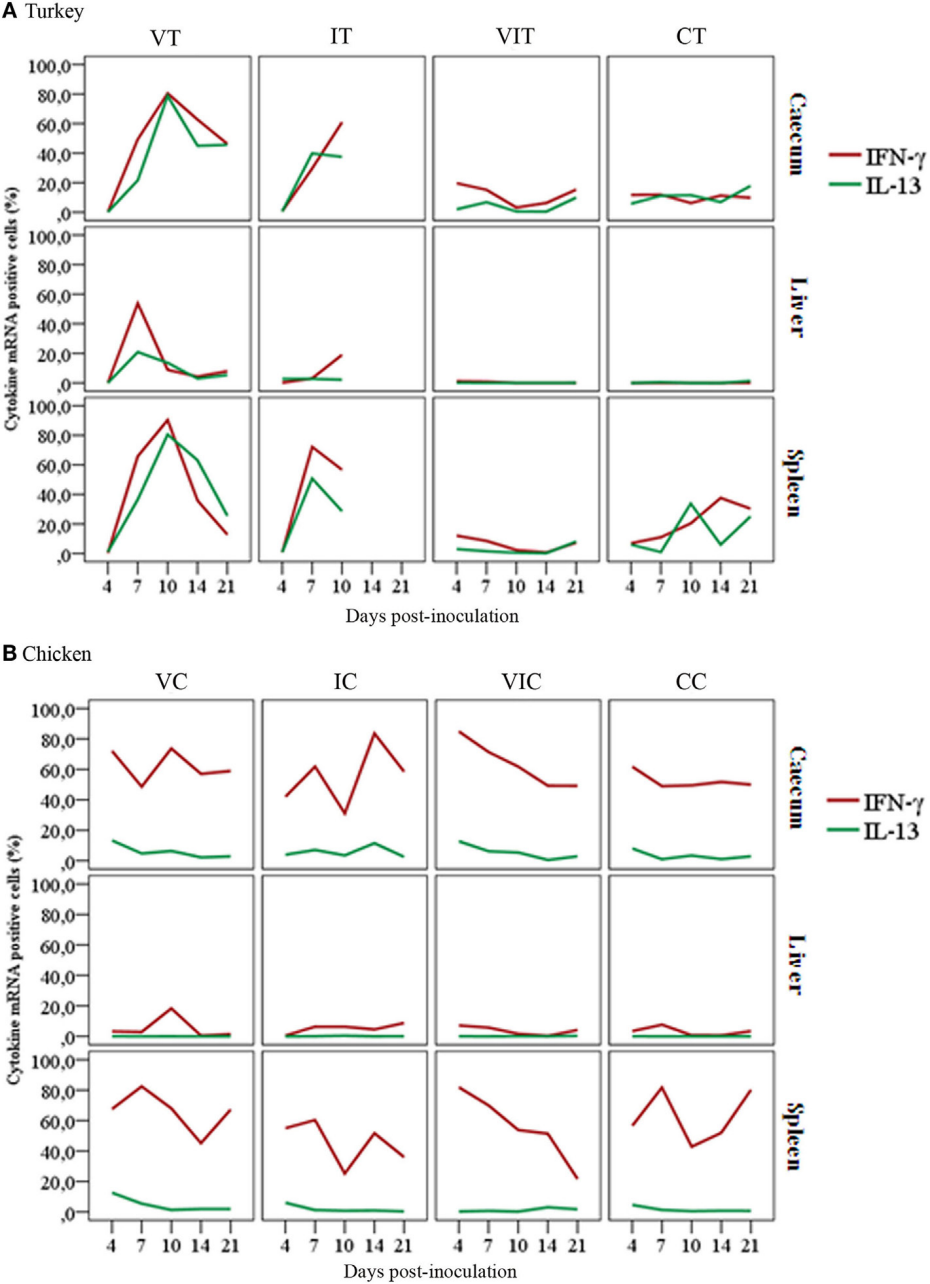
Comparison of cytokine mRNA positive cells show a varying magnitude in the abundance of cells expressing mRNA of interferon gamma (IFN-γ) or interleukin (IL)-13. Panel **(A)** shows the percentage of turkey IFN-γ mRNA positive cells was comparable with IL-13 mRNA positive cells within the respective tissue types, regardless of their treatment condition with slight variations at different days post-inoculation. As indicated in panel **(B)**, the overall percentage of chicken IFN-γ mRNA positive cells was higher compared to the percentage of IL-13 mRNA positive cells in cecum and spleen of the respective groups throughout the trial. Remarkable differences can also be seen in the relative number of IFN-γ mRNA positive cells between cecum of control birds, turkeys (CT) and chickens (CC), with *n* = 3 birds/day.

Comparison of percentages of cytokine mRNA positive cells highlighted a varying magnitude in the abundance of cells expressing mRNA of IFN-γ or IL-13 pertinent to the inoculum, tissue type and host species. In turkeys (Figure 6A), there was a comparable level of IFN-γ and IL-13 mRNA positive cells within each type of tissue independent of the treatment group. Generally, spleen had the highest percentage of IFN-γ and IL-13 mRNA positive cells, followed by cecum whereas liver had the least percentage.

In contrast to turkeys, chicken tissues showed a divergent percentage of IFN-γ and IL-13 mRNA positive cells. Chicken IFN-γ mRNA positive cells were found to be more abundant than IL-13 mRNA positive cells in cecum and spleen of all the experimental groups (Figure 6B). Both tissues had more IFN-γ and IL-13 mRNA positive cells than liver. In contrast to cecum and spleen, a balanced percentage of IFN-γ and IL-13 mRNA positive cells was detected in livers of chickens, with an exception of the slight increment IFN-γ mRNA positive cells at 10 DPI in livers of VC.

Comparing percentages of cytokine mRNA positive cells between tissues of turkeys and chickens also revealed variations in the percentages of positive cells. The most obvious differences were observed in IFN-γ mRNA positive cells in the cecum and spleen of VIC and CC when compared with their counterpart turkey groups (VIT and CT).

### Changes in Immune Cell Population in Chickens During Vaccination or Infection

**Figure 7:**
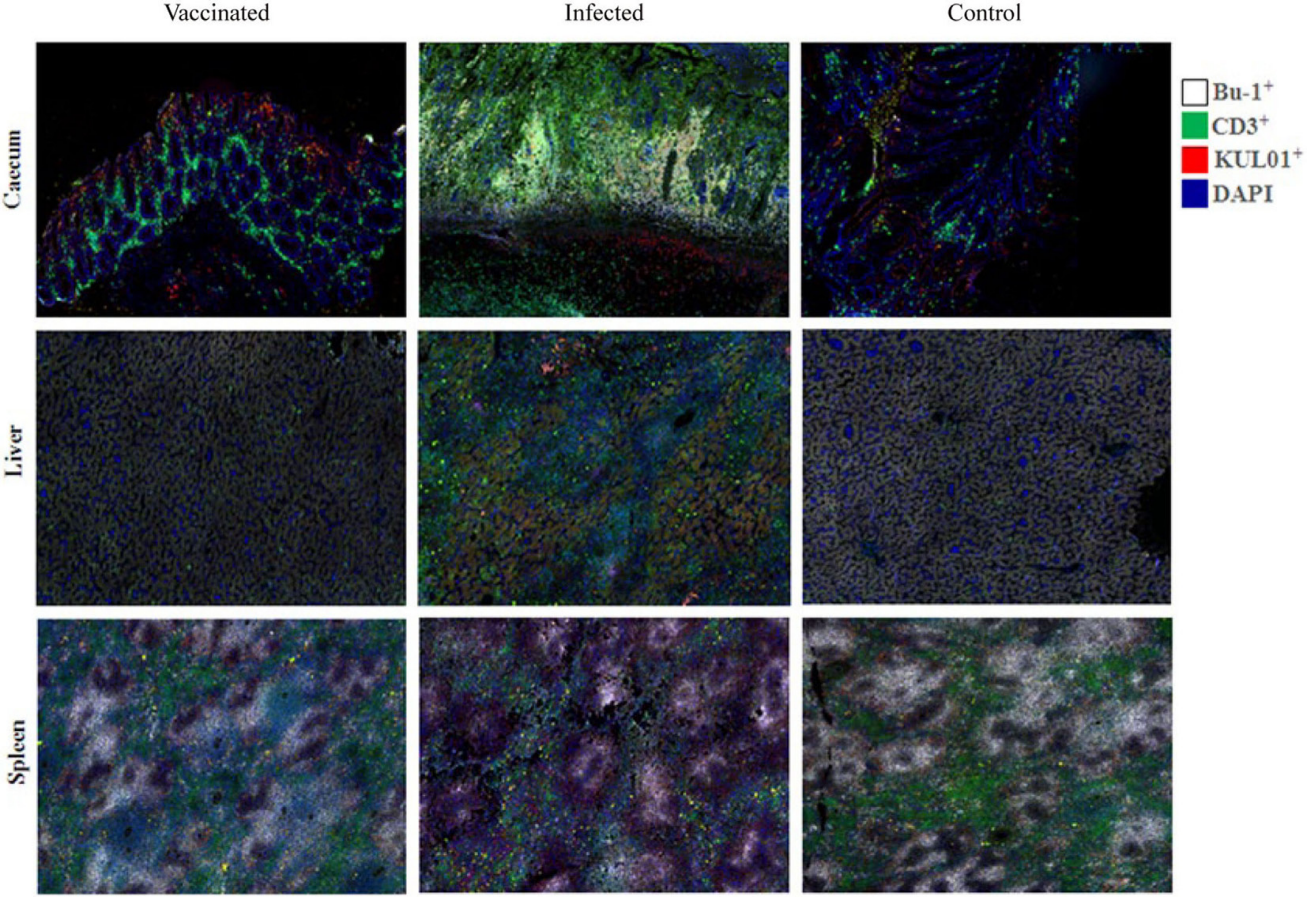
B cells (Bu-1^+^), T cells (CD3^+^) and monocytes/macrophages (KUL01^+^) detected by immunofluorescence in tissues of chickens. An increase of B and T cells in the mucosa of the cecum [4 days post-inoculation (DPI)] and the liver (14 DPI) of infected chickens (inoculated with only virulent histomonads) is indicated by a higher intensity of the respective fluorochromes. By contrast, fluorescent signals of the lymphocytes in the spleen (14 DPI) are comparatively reduced in the same group.

**Figure 8:**
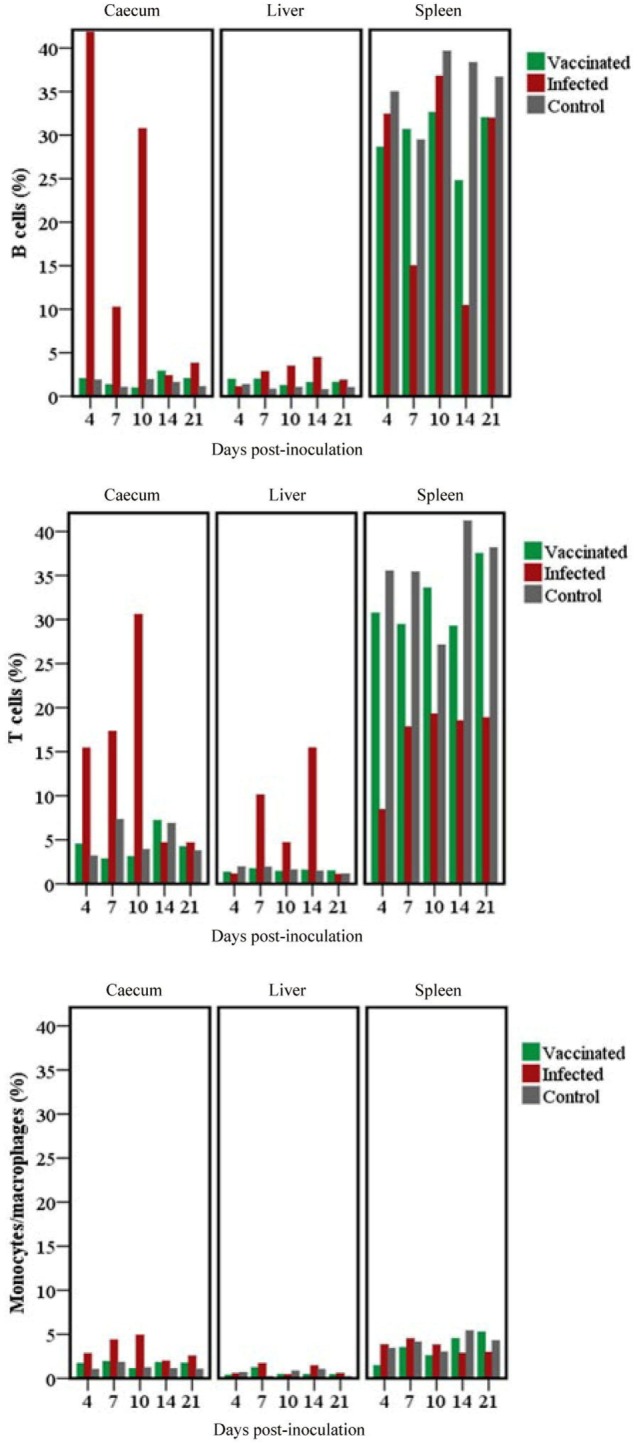
Changes in B cells, T cells and monocytes/macrophages stained in cecum, livers and spleens of chickens. Analysis of the relative number of target cells was performed using TissueQuest after an automated image acquisition with TissueFAXS in tissues of groups of chickens inoculated with only attenuated (vaccinated) or virulent (infected) *Histomonas meleagridis*. Samples were drawn from a single chicken per group for the different days post-inoculation.

B cells, T cells and monocytes/macrophages stained by IF in the cecum, liver and spleen cryosections of chickens from trial 2 inoculated with either virulent or attenuated histomonads revealed changes in the population of these cells at different days post-inoculation (Figure 7). Quantification of the stained cells (Figure 8) showed distinct changes in IC which had an increased percentage of B cells, T cells and monocytes/macrophages in cecum at 4, 7 and 10 DPI as wall as in the liver at 10 and 14 DPI. By contrast, spleens of the same group showed decrement of B cells at 7 and 14 DPI and T cells from 4 DPI until 21 DPI. The levels in VCs were very similar to the control.

### Fold Changes in the Expression Levels of IFN-γ and IL-13 mRNA Measured by RT-qPCR

Expression levels of IFN-γ and IL-13 mRNA were measured from samples collected at 10 and 21 DPI to supplement results obtained by quantification of the expressing cells. In most cases, results of RT-qPCR harmonized with quantification of cells.

**Figure 9:**
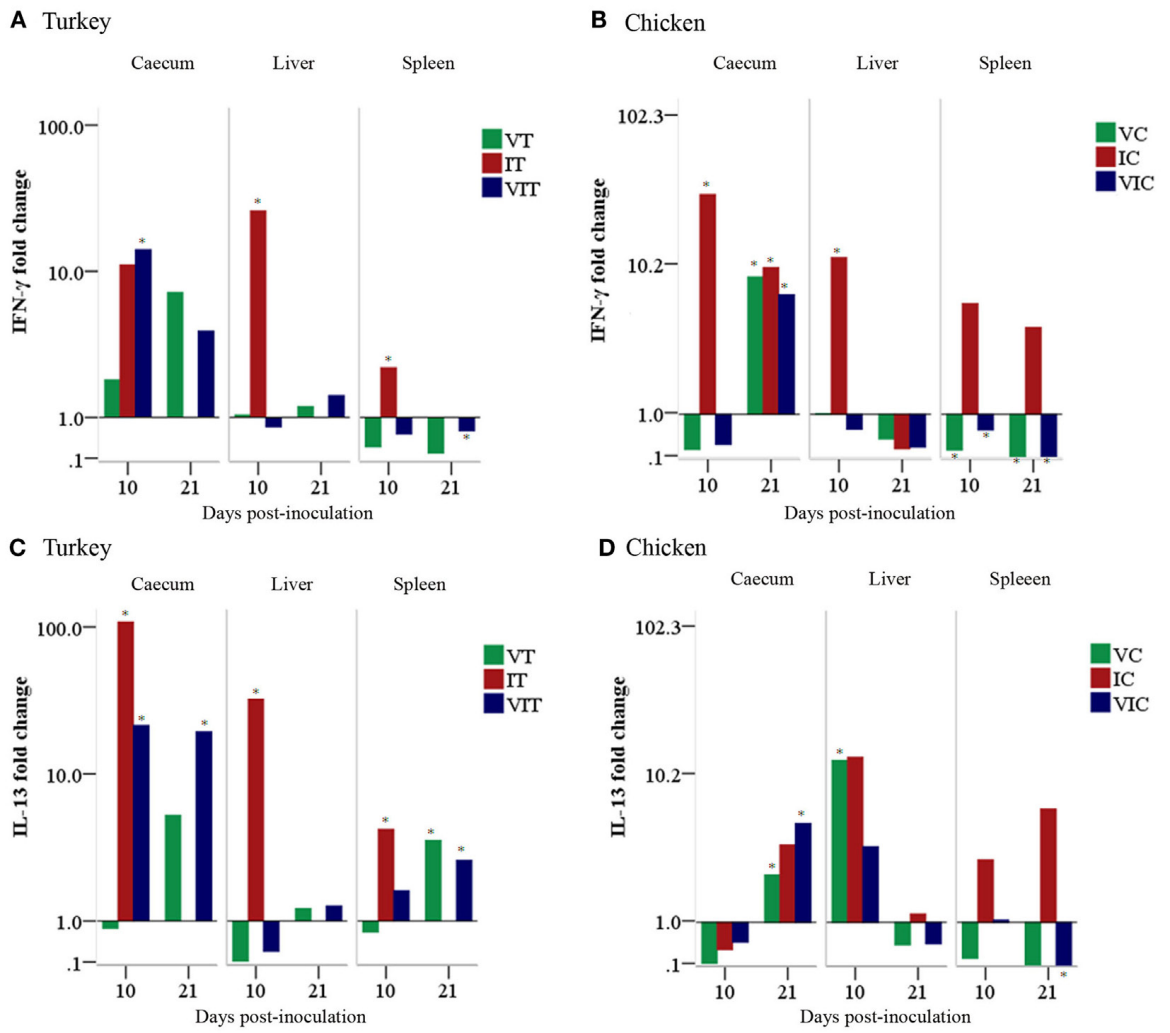
Cytokine mRNA expression levels measured by reverse transcription-qPCR. The expression level of interferon gamma (IFN-γ) in tissues of turkeys **(A)** and chickens **(B)** as well as the expression level of interleukin (IL)-13 in tissues of turkeys **(C)** and chickens **(D)** are shown as a fold change of mRNA expression levels relative to age-matched controls. *n* = 3 birds/day, *P < 0.05.

In turkeys, cecal IFN-γ mRNA (Figure 9A) was upregulated in all of the inoculated groups sampled at 10 and 21 DPI although the only statistically significant change was noticed in caeca of VIT at 10 DPI. Similarly, a significant fold change was found at 10 DPI in liver and spleen of IT. By contrast, the expression was significantly downregulated at 21 DPI in spleens of VIT. In chickens (Figure 9B), cecal IFN-γ mRNA was significantly upregulated in IC at 10 DPI and in all groups at 21 DPI. Livers of IC also had a significantly enhanced expression at 10 DPI, whereas the changes in the other groups were lower. The expression was significantly downregulated in spleens of VC and VIC at 10 and 21 DPI.

Turkey IL-13 mRNA (Figure 9C) transcripts were significantly upregulated in the cecum of IT and VIT at 10 DPI. Similarly, an enhanced expression was found at 21 DPI in VT and VIT. In livers, a significant increment of expression was found in IT sampled at 10 DPI. In spleens, IL-13 transcripts showed increased fold changes at 10 DPI in IC and at 21 DPI in VT and VIT. In chickens, IL-13 expression (Figure 9D) was downregulated at 10 DPI in all caeca samples drawn from inoculated groups although the changes were not significant. The downregulation projected into a significant upregulation of the transcript at 21 DPI in all the groups with significant changes observed in VC and VIC. In livers, a significantly enhanced expression was observed at 10 DPI in VC. In spleen, the transcript was significantly downregulated in VIC at 21 DPI.

## Discussion

The present study quantified cells that express the mRNA of representative cytokines of type 1 and type 2 immune responses, IFN-γ, respectively, IL-13 in cecum, liver and spleen samples for elucidating cytokine expression patterns that could be attributed to vaccination or infection of turkeys and chickens with clonal cultures of *H. meleagridis*. Groups of birds were inoculated with virulent or attenuated histomonads and necropsied at different time points post-inoculation. The time points were selected based on a previous study in which cecal lesions in affected chicken reached the maximum on days 7–14 after infection and then regresses at 21 DPI (21).

Previously, the safety of the vaccine has been tested by administering 10^4^ cultured histomonads exclusively *via* the oral route for turkeys and with an additional dose of 10^4^ parasites cloacally to chickens. Challenges were tested in turkeys with 10^4^ parasites administered by the cloaca route only (5–7). The birds in the main experiment of this study were inoculated with a 3 × 10^5^ histomonads administrated both orally and cloacally which proved the tentative vaccine safe as noticed in vaccinated hosts together with the capability of inducing a potent immunity during challenge.

The distribution of attenuated histomonads was confined to the cecum in the majority of the samples investigated by ISH, which was in agreement with previous findings (6). On the other hand, virulent histomonads migrated further to the livers of all unprotected turkeys were a much more pronounced invasion as compared to livers of unprotected chickens. The differential kinetics in dissemination patterns of virulent histomonads in livers of turkeys and chickens was comparable to a previous study (12). However, the absence of histomonads in the spleen of all experimental birds was in contrast to a previous finding that demonstrated virulent parasites by ISH in a few spleen sections (27). This could be due to the higher dose of histomonads used in the past study which was 19 times higher per bird than in the present study.

This study employed a simultaneous assessment of localization of cytokine mRNA expressing cells and their quantitative differences in response to attenuated and virulent histomonads. Results showed that IFN-γ mRNA positive cells increased in the cecum of vaccinated turkeys without compromising the integrity of the tissue. By contrast, virulent histomonads caused tissue destruction along with the exacerbated increment of positive cells after a decrement of positive cells at 4 DPI. The low percentage of positive cells at this early stages in IT was in agreement with the downregulation observed by Powell et al. (12) who measured the transcript via RT-qPCR. In the present study, no data on RT-qPCR of cytokine expression were determined which limits the direct comparison. However, the authors of the last mentioned study observed a delay in cytokine response of ITs at 3 DPI which later increased to a storm of cytokines after the parasite could migrate to the liver leading to destruction of cecum and liver tissues. In concordance with this, findings of the present work showed that the attenuation of the protozoa allowed the host to project the appropriate immune response without inducing tissue damage. Hence, an effective stimulation of cytokine expression that does not result in tissue damage can be considered as a desirable trait of vaccination with *in vitro* attenuated *H. meleagridis*.

In general, vaccination induces a restricted immune response to develop appropriate local and/or systemic immunity. In the present experiment, vaccinated and challenged turkeys showed a slight increment of IFN-γ mRNA positive cells in the caeca at 4 DPI. This finding indicates that the vaccine provided priming of the immune system against invading virulent histomonads. The fact that the response was shortly activated at the site of infection argues for the presence of effector memory T cells as this cell population is known to counteract re-infection by secreting anti-microbial cytokines such as IFN-γ and TNF-α (28).

The absence of any distinct separation in the relative levels of IFN-γ and IL-13 mRNA expressing cells in turkeys suggests that both type 1 and type 2 immune responses have been elicited during vaccination or infection. This could be due to the protozoa’s mixed host-cellular niche. Even though it is known as an extracellular protozoan, immunohistochemical localization of the parasite in turkey liver revealed that it could occasionally be detected within host cells, mostly phagocytized by macrophages and giant cells (29). Previously, a strict intracellular pathogen (Newcastle disease virus) and extracellular helminth, *Ascaridia galli*, have been shown to cause a polarized type of cytokine expression in chickens (30).

In contrast to turkeys, a higher percentage of IFN-γ mRNA positive cells than IL-13 mRNA were noticed in chickens, regardless of the treatment conditions. Past studies on infection with virulent *H. meleagridis* or dual infection with the cecal nematode, *Heterakis gallinarum*, delivered inconclusive results in regards to type 1/type 2 responses in chicken cecal tonsils or cecum. Quantification of IFN-γ or IL-13 mRNA by RT-qPCR in cecal tonsils of turkeys and chickens infected with *H. meleagridis* inoculum prepared from an infected tissue homogenate showed a development of type 2 response with IL-13 expression although other cytokines including IFN-γ were also enhanced (12). By contrast, a dominance of type 1 response with augmented expression of IFN-γ was noticed in chicken cecum dually infected with *H. meleagridis* and *H. gallinarum* (18). Our data on the predominance of IFN-γ in chicken tissues in response to clonal cultures of histomonads was in agreement with the gene expression studies of dual infections by Schwarz et al. (18), highlighting a type 1 response in this species. Chickens probably enhance IL-13 expression only upon the encounter of the relevant pathogen. Expression of IL-13 in this species, compared to the turkeys, could be stricter, particularly in non-lymphoid tissues (31, 32). Thus, factors such as mRNA decay rate and an inter species difference in the regulation of both cytokines should be considered and explored further for defining a threshold of measurement.

In mammals, the balance between Th1/Th2 lymphocytes subsets, the main sources of signature cytokines of type 1/type 2 immunity, determines susceptibility to some disease states (16). In this context, the presence of a higher percentage of IFN-γ mRNA positive cells in the avian cecum seems to correlate with protection against histomonosis as noticed in chickens and vaccinated turkeys. In a study focusing on an extracellular intestinal protozoan parasite of humans, *Entameba histolytica*, it was demonstrated that asymptomatic carriers of the pathogen had higher levels of IFN-γ, reflecting a Th1 response, while patients showing disease displayed higher levels of IL-4, resembling that of a Th2 response (33). The study further revealed that re-infection in vaccinated mice was mediated by IFN-γ-producing CD4^+^ T cells and IL-17 secreting CD8^+^ T cells. Overall, in the mentioned study, IFN-γ was shown to enhance anti-parasitic activity in macrophages, which might be as well an important mechanism in the immune response against histomonosis of poultry. It is worth mentioning here that the mammalian key type 2 cytokine, IL-4, has been shown in the avian immune system to be less expressed compared to IL-13 in several extracellular infection models (12, 18, 23, 30, 34), hence measuring IL-13 was opted in the current study.

An important observation in the actual study was the difference in the percentage of IFN-γ mRNA positive cells in CC compared to CT. Reasons to this could be diverse and would demand research based verification. It can, however, be speculated that growth-selected turkey lines might have a trade-off between immune function and growth. Previous metanalysis on various poultry lines selected for increased growth showed a strong and significant decrease in immune function (35). In this regard, the higher surface-area abundance of these cells in the chicken cecum might be one of the reasons why this species is less susceptible to histomonosis. Their well-equipped caeca might make them immunologically competent to ward-off the protozoa at the port of entry. With this observation, it can be assumed that the presence of IFN-γ in cecum is supportive in the defense against histomonads as was shown enhancing resistance against coccidiosis (36) and *E. coli* (37) challenge. Even though further studies are needed to elucidate the specific effector function of this enhanced percentage of cells during vaccination or infection with histomonads, several effects of the cytokine might play a role in the immune response against the parasite. IFN-γ is known to promote cell-mediated immunity through activation of macrophages for phagocytosis (17). Moreover, it has been shown to stimulate secondary IgG response and enhance the expression of the genes for MHC class II in chickens (38). These traits can endow the hosts with an immune response that can better present histomonad antigens as well as efficiently fight infection.

Avian IFN-γ can be presumably be produced by macrophages, dendritic cells, CD4^+^ and CD8^+^ T cells, like in mammals (39–42). Recently, IFN-γ producing innate B lymphocytes have also been characterized in mice that produce high levels of IFN-γ that promotes macrophage activation, facilitating the innate immune response during early stages of infection (43). Likewise, various innate and adaptive stages of immune cells can synthesize IL-13. Hematopoietic cells of the innate immune system such as eosinophils, basophils and mast cells have been shown to produce IL-13 in mice (44). In addition, Th2 (CD4^+^) cells are described as the main source of IL-13 during the effector phase of type 2 immune responses in worm-infested mice (45). In this study, the cellular background of the cytokine mRNAs could not be directly demonstrated due to the lack of immune cell markers for turkeys and the incompatibility of the staining techniques. However, interpretation of the IF results in context of the cytokine ISH results in chickens indicate that more cell types than B cells, T cells and macrophages could be involved in producing the cytokines as the percentages of cytokine-expressing cells were higher than the immune cells quantified by IF. Nevertheless, this indication needs to be verified in prospective studies by establishing a technique enabling co-staining of cytokines and cell markers.

This study employed RT-qPCR on samples taken from selected days to observe the congruence of mRNA quantification results with that of quantification of ISH stained cells. The high percentage of IFN-γ mRNA positive cells in the cecum of VT and IT coinciding with an enhanced expression of IFN-γ mRNA at 10 DPI demonstrated the agreement of the results. By contrast, some discrepancies were observed for instance with the increased percentages of IFN-γ mRNA positive cells in liver and spleen of VT in comparison to the RT-qPCR results of both tissues at 10 DPI. The discrepancies may arise from the difference in the detection system of both techniques. ISH labels mRNA positive cells and a higher percentage indicates a wider distribution of positive cells in a given area, whereas PCR amplifies a pooled mRNA extracted from a myriad of cells which may have more or no expressed transcript. Hence, a result of a high percentage of positive cells with a low or moderate amount of the transcript might be underestimated by fewer numbers containing a higher quantity of the transcript and *vice versa* (46).

In conclusion, immunization of turkeys using *in vitro* attenuated *H. meleagridis* causes an increased percentage of IFN-γ and IL-13 mRNA positive cells in the cecum of turkeys without compromising the integrity of the tissue. Infection in non-immunized turkeys also induced an increased presence of cytokine mRNA positive cells in cecum following an early decrement but with severe destruction of the mucosa, as well as infiltration of cytokine-expressing cells up to the muscularis layer with similar destruction and cytokine distribution in livers. Overall, the cytokine expression pattern in turkeys followed both type 1 and type 2 cytokine response with attenuated and virulent histomonads. The higher percentage of IFN-γ mRNA positive cells in chicken cecum than turkeys might also be implicated in the differential survival of this species during histomonosis. Consequently, since a higher percentage of IFN-γ mRNA positive cells have been observed in chickens as well as in immunized turkeys capable to defend histomonosis with early expression of IFN-γ mRNA, it can be concluded that increased presence of IFN-γ mRNA positive cells indicates a protective trait against histomonosis.

## Ethics Statement

The animal trials were discussed and approved by the institutional ethics committee and the national authority according to §26 of the Law for Animal Experiments, Tierversuchsgesetz 2012—TVG 2012, license number: bmwf GZ 68.205/0147-II/3b/2013.

## Author Contributions

DL, MH and FK conceived and designed the work. FK, TM, DL and PW performed the animal trial and collection of samples. FK and PW performed ISH and IF experiments. FK and DL performed image acquisition and analysis. TM performed and analyzed the RT-qPCR. FK and DL interpreted the data and drafted the manuscript. MH revised the manuscript critically for important intellectual content. All authors read and approved the final manuscript.

## Conflict of Interest Statement

The authors declare that the research was conducted in the absence of any commercial or financial relationships that could be construed as a potential conflict of interest.

## Supplementary Material

Figure S1 Quantification strategy for cytokine mRNA positive cells detected by bright field microscopy and TissueFAXS image acquisition. The image is given exemplary from a single region of interest in cecum, liver and spleen tissue sections after *in situ* hybridization (ISH) staining with turkey interferon gamma antisense probe. Original bright field microscopy images **(A)**, setting a cutoff value to discriminate ISH (NBT-BCIP) positive cells **(B)** and backward viewing **(C)** to verify the correct discrimination of ISH positive (red mask) and negative cells (green mask) of the source image.

Figure S2 Attenuated histomonads localized by *in situ* hybridization in the cecum of turkeys necropsied at 10 days post-inoculation showing the parasites restricted to the lumen of the cecum.

## Abbreviations

CC, control chickens; CT, control turkeys; DIG, digoxigenin; DPI, days post-inoculation; IC, infected chickens; IF, immunofluorescence; IFN-γ, interferon gamma; IL, interleukin; ISH, *in situ* hybridization; IT, infected turkeys; MLS, mean lesion score; Th, T helper; VC, vaccinated chickens; VIC, vaccinated and infected chickens; VIT, vaccinated and infected turkeys; VT, vaccinated turkeys.
