# Supplementary figures and images for: Allocation of Interferon Gamma mRNA Positive Cells in Caecum Hallmarks a Protective Trait Against Histomonosis
Source: Front Immunol. 2018 May 28;9:1164. doi: 10.3389/fimmu.2018.01164 (PMC5985309; doi:10.3389/fimmu.2018.01164)

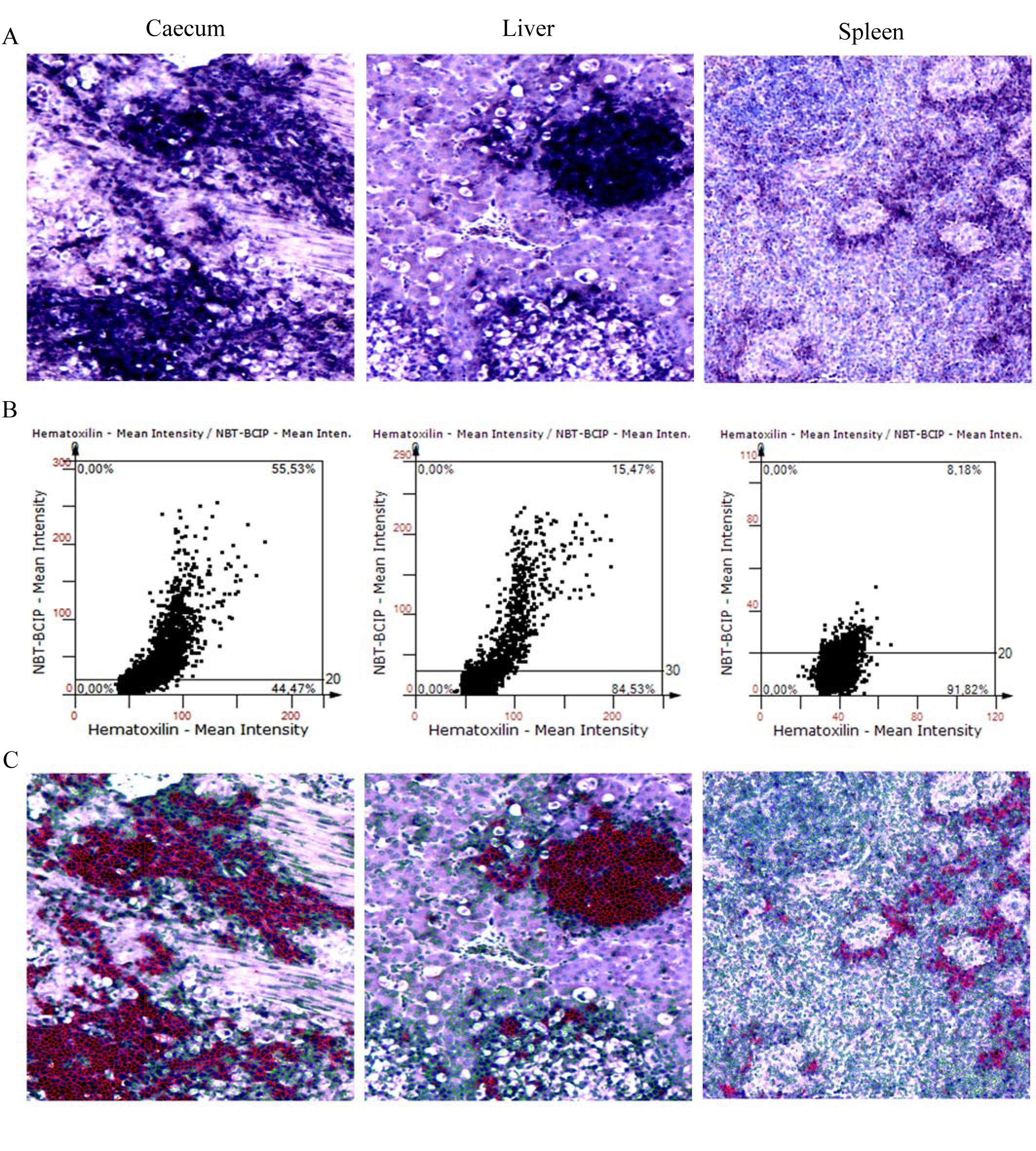

Supplement: Figure S1 — Quantification strategy for cytokine mRNA positive cells detected by bright field microscopy and TissueFAXS image acquisition. The image is given exemplary from a single region of interest in cecum, liver and spleen tissue sections after in situ hybridization (ISH) staining with turkey interferon gamma antisense probe. Original bright field microscopy images (A), setting a cutoff value to discriminate ISH (NBT-BCIP) positive cells (B) and backward viewing (C) to verify the correct discrimination of ISH positive (red mask) and negative cells (green mask) of the source image. [file Image_1.JPEG]

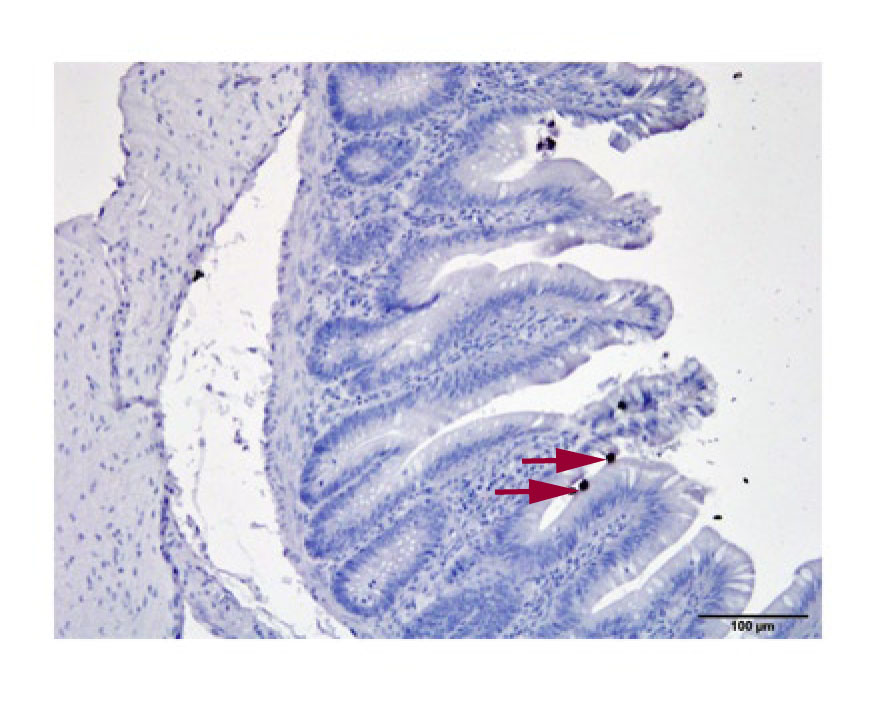

Supplement: Figure S2 — Attenuated histomonads localized by in situ hybridization in the cecum of turkeys necropsied at 10 days post-inoculation showing the parasites restricted to the lumen of the cecum. [file Image_2.JPEG]
